# Non-Standard Diagnostic Assessment reliability in psychiatry: A study in a Brazilian outpatient setting using Kappa

**DOI:** 10.1192/j.eurpsy.2024.977

**Published:** 2024-08-27

**Authors:** H. Rocha Neto, D. Telles-Correia, L. Koiller, M. Cavalcanti

**Affiliations:** ^1^Psiquiatria, Faculdade de Medicina de Lisboa, Lisbon, Portugal; ^2^Programa de Pós Graduação em Psiquiatria e Saúde Mental, Universidade Federal do Rio de Janeiro, Rio de janeiro, Brazil

## Abstract

**Introduction:**

The use of Structured Diagnostic Assessments (SDAs) is a solution for unreliability in psychiatry and the gold standard for diagnosis. However, except for studies between the 50s and 70s, reliability without the use of Non-SDAs (NSDA) is seldom tested, especially in non-Western, Educated, Industrialized, Rich, and Democratic (WEIRD) countries.

**Objectives:**

We aim to measure reliability between examiners with NSDAs for psychiatric disorders.

**Methods:**

We compared diagnostic agreement after clinician change, in an outpatient academic setting. We used inter-rater Kappa measuring 8 diagnostic groups: Depression (DD: F32, F33), Anxiety Related Disorders (ARD: F40–F49, F50–F59), Personality Disorders (PD: F60–F69), Bipolar Disorder (BD: F30, F31, F34.0, F38.1), Organic Mental Disorders (Org: F00–F09), Neurodevelopment Disorders (ND: F70–F99) and Schizophrenia Spectrum Disorders (SE: F20–F29) (Check table 1 about diagnosis hyerarchy and observed frequency in sample). Cohen’s Kappa measured agreement between groups, and Baphkar’s test assessed if any diagnostic group have a higher tendency to change after a new diagnostic assessment. This research was approved by IPUB’s ethical committee, registered under the CAAE33603220.1.0000.5263, and the UTN-U1111-1260-1212.

**Results:**

We analyzed 739 reevaluation pairs, from 99 subjects who attended IPUB’s outpatient clinic. Overall inter-rater Kappa was moderate, and none of the groups had a different tendency to change (Check table 2 for diagnostic change distribution). Our tests achieved the followinf results: Cohen Kappa 0.70, IC: 0.66– 0.74; Weighted Kappa 0.72, IC:0.72 – 0.72; Bhapkar Test X² = 5.98, Df = 7, P-value = .55; Achieved Power (w=0.1): 0.93

**Table 2:** Agreement between examiners for eight diagnostic groups

	ARD	BD	DD	DRD	ND	Organic	PD	SSD
ARD	**39**	3	9	0	2	0	3	3
BD	1	**154**	7	3	2	2	4	10
DD	9	10	**71**	0	0	2	5	9
DRD	0	2	0	**4**	0	0	0	2
ND	1	2	1	0	**51**	1	1	6
Organic	0	1	0	0	3	**20**	0	5
PD	4	2	1	1	0	0	**33**	3
SSD	5	20	11	2	8	5	4	**192**

**Conclusions:**

NSDA evaluation was moderately reliable, but the lack of some prevalent hypothesis inside the pairs raised concerns about NSDA sensitivity to some diagnoses. Diagnostic momentum bias (that is, a tendency to keep the last diagnosis observed) may have inflated the observed agreement.

**Disclosure of Interest:**

None Declared

